# The complete chloroplast genome of *Paeonia* cv. Hwang-Moran (Paeoniaceae)

**DOI:** 10.1080/23802359.2020.1860721

**Published:** 2021-01-27

**Authors:** Jinsu Gil, Jin Hee Park, Ki Hoon Yoon, So Hyun Park, Minjee Lee, Yi Lee

**Affiliations:** a Department of Industrial Plant Science and Technology, Chungbuk National University, Cheongju, South Korea; b Nakdonggang National Institute of Biological Resources, Sangju, South Korea; c Research Institute, Soleon Co., Ltd, Cheongju, South Korea; d Green Plant Institute, Yongin, South Korea

**Keywords:** Chloroplast genome, next-generation sequencing, *Paeonia* cv. Hwang-Moran, phylogenetic tree, yellow flower

## Abstract

The complete chloroplast genome of *Paeonia* cv. Hwang-Moran (PHM), a yellow flowering tree peony, was *de novo* assembled and characterized from high-throughput next-generation sequencing data. The total length of the circular PHM chloroplast genome was 152,519 bp, including a large single-copy (LSC) region of 84,214 bp, a small single-copy (SSC) region of 17,026 bp, and a pair of inverted repeat regions (IRs) of 25,640 bp. The entire chloroplast genome contained 111 genes, including 77 protein-coding genes, 30 tRNAs, and four rRNAs. A phylogenetic tree constructed using the PHM and related chloroplast genome sequences revealed its close taxonomic relationship with *P. ludlowii* within the genus *Paeonia*.

*Paeonia* is an important medicinal and ornamental plant. The yellow flower color of the tree peony *Paeonia* cv. Hwang-Moran (PHM) is unlike that of common peony flowers. This characteristic makes PHM especially desirable for use as an ornamental plant (Zhou et al. 2014; Zhao et al. 2016). *Paeonia* plants have been used for their anti-aggregatory and immunomodulatory activity and for sun protection and skin whitening (Koo et al. 2010; Lin et al. 2019; Ma et al. 2019). PHM is an important material for studying the genetic diversity, phytogeography, and evolution of the *Paeonia* and for developing new tree peony varieties.

In the present study, we generated the complete chloroplast genome sequence of PHM. We collected a fresh leaf from PHM in an experimental field in Chungbuk National University (36°37′28.61″N, 127°27′18.36″E). The dried plant specimen (NIBRVP0000634106) was deposited at the Herbarium of the National Institute of Biological Resources, Incheon, Korea. Genomic DNA was isolated from the sample using a DNeasy Plant Mini Kit (Qiagen, Valencia, CA) and used to prepare an Illumina paired-end genomic library with an average size of 661 bp on the Illumina HiSeq 2500 platform by Theragen (Suwon, South Korea) following the manufacturer’s protocol. A total of 87.6 Gbp high-throughput 150 base-pair PE reads were obtained and assembled using CLC Genomics Workbench (ver. 7.5, CLC Inc., Aarhus, Denmark). The resulting chloroplast genome was annotated with the DOGMA program (Wyman et al. 2004) and manually curated based on BLAST searches.

The complete chloroplast genome of PHM (GenBank accession no. MK860970) was a quadripartite circular structure of 152,519 bp in size. The genome contained a pair of identical inverted repeat (IRa and IRb) regions of 25,640 bp, which were separated by a large (84,214 bp) single-copy (LSC) region and a small (17,026 bp) single-copy (SSC) region. The overall GC content of the PHM chloroplast genome was 38.44%, and the GC contents of the LSC, SSC, and IR regions were 36.7, 32.7, and 43.1%, respectively. This genome contained 111 genes, including 77 protein-coding genes, 30 tRNA genes, and four rRNA genes.

We performed phylogenetic analysis using the complete chloroplast genome sequence of PHM and those of 16 species, including 13 ingroup species of the Saxifragales, to which *Paeonia* belongs, and three outgroup species of the Vitales. To generate the tree, we used the General Time Reversible nucleotide substitution model (Tavare 1986) and the maximum-likelihood algorithm with 1000 bootstrap replications in CLC Genomics Workbench. In this tree, seven taxa of Paeoniaceae formed a monophyletic group separate from those of seven other species of the Saxifragales (Figure 1). The phylogenetic tree placed PHM close to *P. ludlowii.*

**Figure 1.**
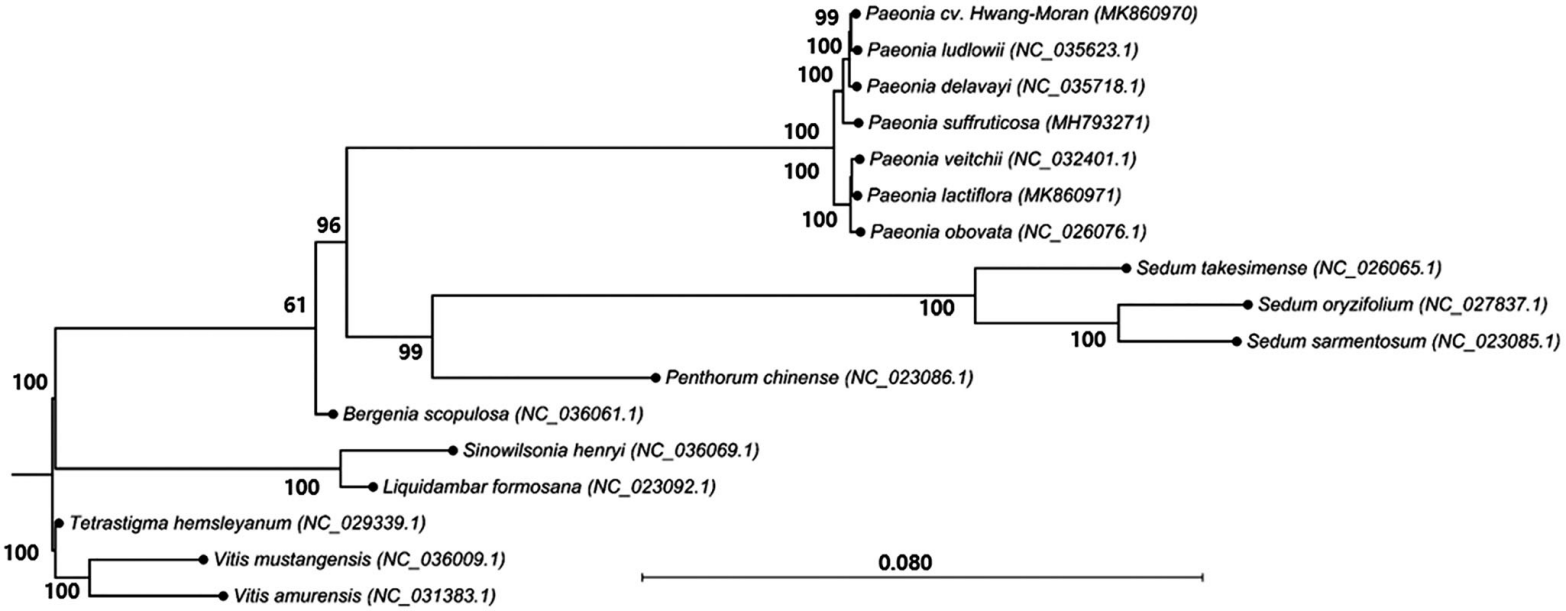
Maximum-likelihood phylogenetic tree of *Paeonia* cv. Hwang-Moran with other species in Saxifragales, to which the Paeoniaceae family belongs. The chloroplast sequences of the Vitaceae family were used as the outgroup. Numbers in the nodes are bootstrap values from 1000 replicates.

## Data Availability

The data that support the findings of this study are openly available in NCBI at https://www.ncbi.nlm.nih.gov/search/all/?term=MK860970, reference number MK860970.
